# Bis(2,6-pyrazolyl)pyridines as a New Scaffold for Coordination Polymers

**DOI:** 10.3390/molecules28114275

**Published:** 2023-05-23

**Authors:** Igor A. Nikovskiy, Pavel V. Dorovatovskii, Valentin V. Novikov, Yulia V. Nelyubina

**Affiliations:** 1 Nesmeyanov Institute of Organoelement Compounds, Russian Academy of Sciences, Vavilova Street 28, 119991 Moscow, Russia; igornikovskiy@mail.ru; 2 National Research Centre “Kurchatov Institute”, Akademika Kurchatova pl. 1, 123182 Moscow, Russia; paulgemini@mail.ru; 3 Moscow Institute of Physics and Technology, National Research University, Institutskiy per. 9, 141700 Dolgoprudny, Russia

**Keywords:** 2,6-bis(pyrazol-3-yl)pyridine, coordination polymer, molecular design, proton-coupled electron transfer, spin crossover

## Abstract

Two coordination polymers, Fe(L^OBF^_3_)(CH_3_COO)(CH_3_CN)_2_]_n_•nCH_3_CN and [Fe(L^O−^)_2_AgNO_3_BF_4_•CH_3_OH]_n_•1.75nCH_3_OH•nH_2_O (L^O−^ = 3,3′-(4-(4-cyanophenyl)pyridine-2,6-diyl)bis(1-(2,6-dichlorophenyl)-1H-pyrazol-5-olate)), were obtained via a PCET-assisted process that uses the hydroxy-pyrazolyl moiety of the ligand and the iron(II) ion as sources of proton and electron, respectively. Our attempts to produce heterometallic compounds under mild conditions of reactant diffusion resulted in the first coordination polymer of 2,6-bis(pyrazol-3-yl)pyridines to retain the core N_3_(L)MN_3_(L). Under harsh solvothermal conditions, a hydrogen atom transfer to the tetrafluoroborate anion caused the transformation of the hydroxyl groups into OBF_3_ in the third coordination polymer of 2,6-bis(pyrazol-3-yl)pyridines. This PCET-assisted approach may be applicable to produce coordination polymers and metal–organic frameworks with the SCO-active core N_3_(L)MN_3_(L) formed by pyrazolone- and other hydroxy-pyridine-based ligands.

## 1. Introduction

Coordination polymers (CPs) and metal–organic frameworks (MOFs) are crystalline materials with a periodic *n*-dimensional structure made of metal ions and organic ligands [1]. Featuring unique (such as permanent porosity [1]) and highly tunable properties, MOFs have found use in sensing [2], catalysis [3], gas storage and separation [4], the applications relying on the delicate control over the adsorption/desorption ability of MOFs towards guest molecules. To achieve this control [5,6], one of the key strategies is to incorporate a switchable component that allows MOFs to undergo a reversible transformation triggered by an external stimulus (light, temperature, pressure or presence of guest compounds) [7]. Such a transformation affects the pore size, so there is no need for high-temperature and low-pressure conditions for desorption of guests [7].

The switchability of MOFs can be achieved by using organic ligands prone to light-induced cis/trans isomerization [8,9], ring opening/closing reactions [10,11] and temperature-induced coil-globule transformation [12], or via radical generation/oxidative emission quenching of europium ion [13], intercalation [14] and spin-crossover phenomenon [11,15,16].

**Scheme 1:**
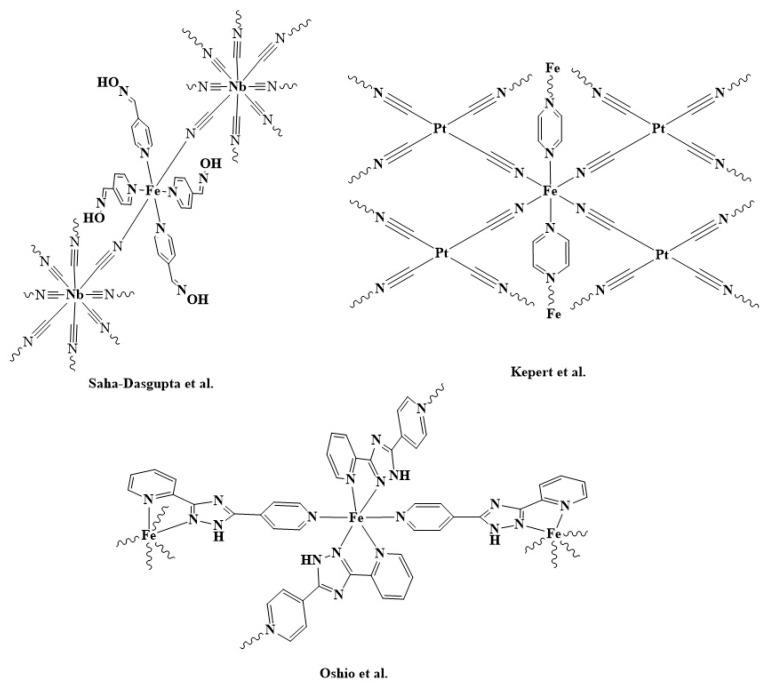
Selected MOFs with (pseudo)octahedral complexes as ligands [19,22,23].

Spin-crossover (SCO) compounds [17] are frequently used for adsorption/desorption control [7] via temperature [18], pressure [19] or guest molecules [20]. A typical way to introduce them into a MOF [7,21] implies a combination of an SCO-active metal ion, mostly iron(II) in a (pseudo)octahedral coordination environment of N-donor ligands [17], with simple organic and inorganic linkers that are easily accessed or commercially available (such as pyrazine and cyanide anions) [15,18,21,22], or with rationally designed polydentate ligands with the desired number of metal ion binding sites [23] (Scheme 1). Unfortunately, it is hard to predict whether an SCO would occur in a CP or a MOF resulted from this ‘self-assembly’ approach [15]. The SCO ability of these crystalline materials strongly depends on the nature of the ligands and their denticity/chelate ring(s) sizes, steric environment around the SCO-active metal ion, topology of the CP or MOF and other factors [24] that are difficult to control [7].

**Scheme 2:**
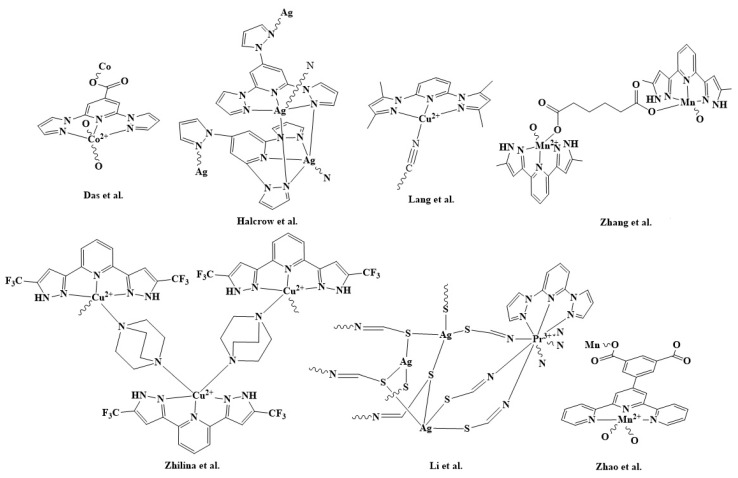
Selected CPs with 1-bpp, 3-bpp and tpy ligands [27,28,30,32,35,38,39].

An alternative pathway towards SCO-active CPs and MOFs is to use (pseudo)octahedral d^4^–d^7^ complexes with the known SCO behavior as ligands [15] with additional coordination sites [25]. To synthesize MOFs or CPs, however, harsh solvothermal conditions are often exploited [25], which may be detrimental to the SCO complexes with low stability towards coordinating solvents, such as DMF and methanol, at high temperatures [26]. For this reason, one of the most studied family of SCO compounds—the (pseudo)octahedral complexes of 2,6-bis(pyrazol-1-yl)pyridines (1-bpp) [17]—never appeared as a ligand with a retained core N_3_(L)MN_3_(L) in a CP or MOF (L = 1-bpp), and neither did the complexes with similar tridentate N-donor ligands, such as isomeric 2,6-bis(pyrazol-3-yl)pyridines (3-bpp) and terpyridines (tpy). These N-donor ligands are, however, known to produce 1D-, 2D- and 3D-CPs of transition metals [27,28,29,30,31,32,33,34,35,36,37] or lanthanides [34,38] with MN_3_(L)X_n_ (*n* = 1, 2, 4, 6; X = additional coordination site of the tridentate ligand L, another ligand or solvent) coordination modes (Scheme 2) for catalysis [33], photovoltaics [32], catalysis [39] and other applications [38]. Most of them include tpy and its derivatives [34]. Of the 1-bpp family, parent 1-bpp [28,36,38], 3,5-dimethyl-substituted 1-bpp [29,30] and 1-bpp with the para-COOH group at the pyridine ring [27] produce heteroleptic and, sometimes, homoleptic CPs. For isomeric 3-bpp, there are only a few examples of heteroleptic 1D-CPs [32,33,37,39] of 5,5′-di-substituted 3-bpp and one, of a homoleptic 1D-CP {[Ag_2_(H_2_L)_2_](NO_3_)_2_•H_2_O}_n_ with silver(I) ion [40] tetracoordinated by two of the same 3-bpp. Unsubstituted NH groups in this ligand are prone to deprotonation in the presence of a base [41], thereby creating an additional coordination site for an SCO-active metal ion [42], which dramatically changes the topology and the SCO activity of the resulting CPs or MOFs.

**Scheme 3:**
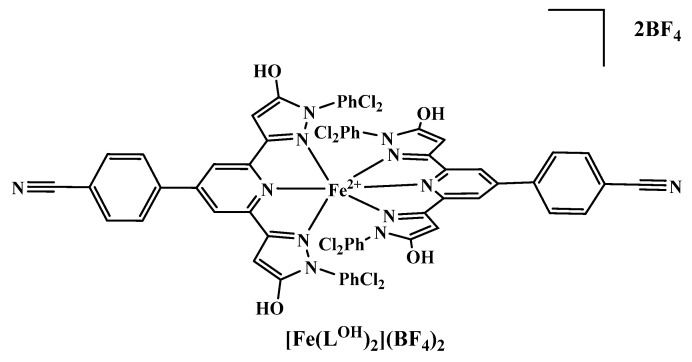
The complex [Fe(L^OH^)_2_](BF_4_)_2_ as a potential linker to produce CPs in this study [44]. PhCl_2_ stands for ortho-dichloro-functionalized N-phenyl groups.

Recently, we proposed a counterintuitive ligand design with ortho-dichloro-functionalized N-phenyl groups to produce the first SCO-active iron(II) complexes of N,N′-disubstituted 3-bpp [43]. Among other things, it allowed us to induce an SCO centered around room temperature [44] by decorating the pyridine moiety of 5,5-dihydroxy-substitited 3-bpp with the p-cyanophenyl group (Scheme 3).

By bonding to another metal ion via this additional coordination site, the homoleptic iron(II) complex [Fe(L^OH^)_2_](BF_4_)_2_ (L^OH^ = 4-(2,6-bis(1-(2,6-dichlorophenyl)-5-hydroxy-1H-pyrazol-3-yl)pyridin-4-yl)benzonitrile) may act as a linker to produce CPs and MOFs. Our attempts to synthesize those with different sources of metal ions as nodes resulted in rare homoleptic CPs to contain the 3-bpp ligand and the first one to retain the core N_3_(L)MN_3_(L) via the proton-coupled electron transfer (PCET) reaction. This reaction, which is mostly found in hydroxo complexes [45] or organic compounds, such as amino-substituted phenols [46], involves the transfer of electrons and protons from one atom to another. It is useful in homolytic activation of X-H (X = S, N, O, C) and C=Y (e.g., Y = O) bonds [47,48,49] and in asymmetric coupling reactions [48,50]. The homoleptic complex [Fe(L^OH^)_2_](BF_4_)_2_ with the hydroxyl groups as the source of the protons may potentially undergo a PCET similar to iron(II) complexes with bidentate imidazole-based ligands [51], although no such examples were yet reported for 1-bpp, 3-bpp or tpy ligands.

## 2. Results and Discussion

To obtain CPs and MOFs by exploiting an additional coordination site of the iron(II) complex [Fe(L^OH^)_2_](BF_4_)_2_, we used two different approaches: reactant diffusion [52] at room temperature and solvothermal synthesis, which is often used to produce high-quality crystals for X-ray diffraction [25,53]. As the sources of the metal ion to serve as a node, different inorganic salts were chosen, such as ZnCl_2_, Zn(OAc)_2_, FeCl_2_, (CH_3_CN)_2_PdCl_2_, AgNO_3_, CuSO_4_, NiCl_2_ and Co(OAc)_2_. They feature a good solubility in methanol, which does not cause the decomposition of the polymeric product.

**Figure 1:**
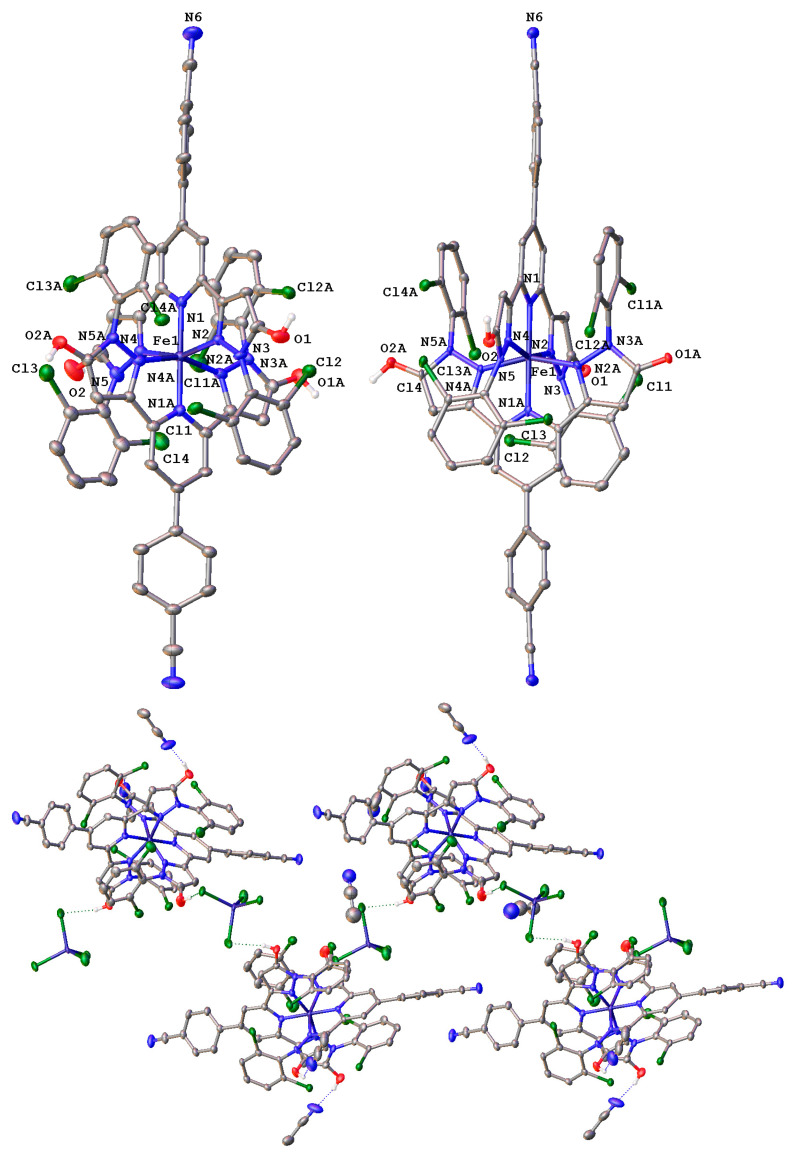

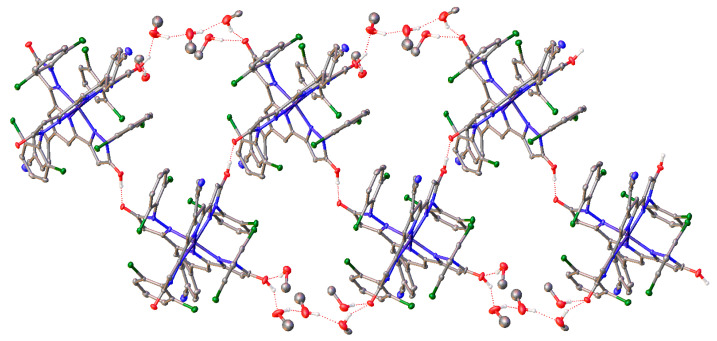
General view of the cation [Fe(L^OH^)_2_]^2+^ in [Fe(L^OH^)_2_][FeCl_4_]•5CH_3_CN (top left), the neutral complex [Fe(L^O−^)_2_] in [Fe(L^O−^)_2_]•5CH_3_OH (top right), fragments of the crystal packing illustrating hydrogen-bonded spirals in [Fe(L^OH^)_2_][FeCl_4_]•5CH_3_CN (middle) and hydrogen-bonded zig-zag chains in [Fe(L^O−^)_2_]•5CH_3_OH (bottom). Hydrogen atoms except those of OH groups are omitted, non-hydrogen atoms are shown as thermal ellipsoids (*p* = 30%), and only labels of the heteroatoms are given.

**Figure 2:**
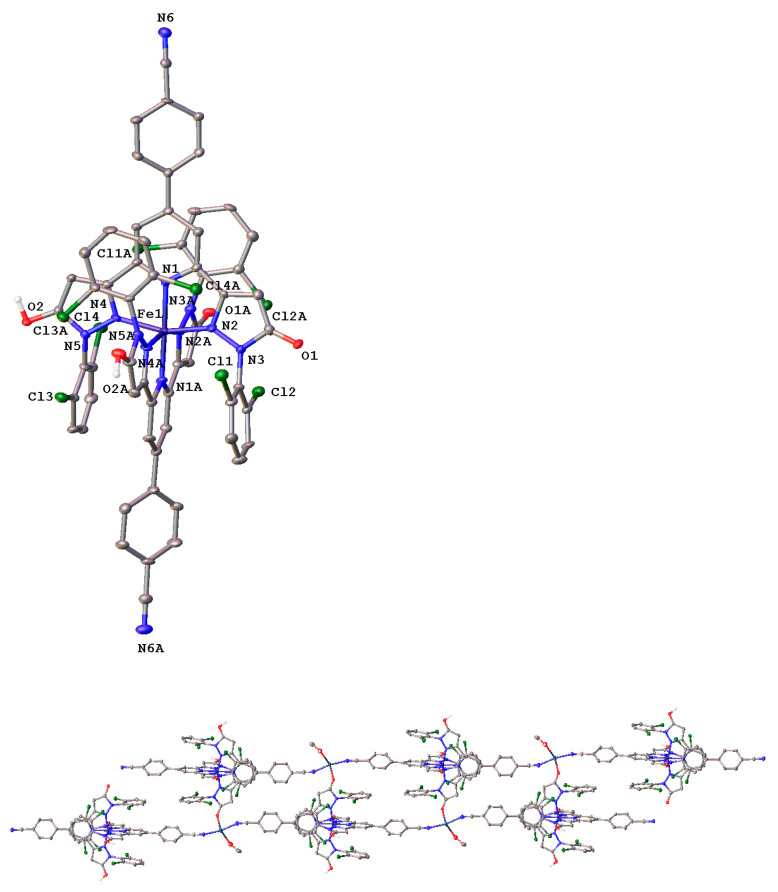
A general view of the complex species [Fe(L^O−^)_2_] (**top**) and a fragment of the 1D-coordination polymer (**bottom**) in [Fe(L^O−^)_2_AgNO_3_BF_4_•CH_3_OH]n•1.75nCH_3_OH•nH_2_O. Nitrate and tetrafluoroborate anions, solvent methanol and water molecules and hydrogen atoms except those of OH groups are omitted, non-hydrogen atoms are shown as thermal ellipsoids (*p* = 30%) and only labels of the heteroatoms in an asymmetric part of the unit cell are given.

Use of the excess of FeCl_2_, CuSO_4_ and AgNO_3_ (10 eq.) under mild conditions of reactant diffusion [52] in methanol to retain the core N_3_(L)MN_3_(L) resulted in non-soluble crystalline products [54]; with other inorganic salts, only a minor color change of the solution was observed. X-ray diffraction of the obtained crystalline products showed them to be molecular complexes of iron(II) [Fe(L^OH^)_2_][FeCl_4_]•5CH_3_CN and [Fe(L^O−^)_2_]•5CH_3_OH (Figure 1) with acetonitrile and methanol as lattice solvents and a 1D-CP of iron(III) [Fe(L^O−^)_2_AgNO_3_BF_4_•CH_3_OH]n•1.75nCH_3_OH•nH_2_O (Figure 2) with methanol as both the co-ligand and lattice solvent. In the latter two cases, one of the OH groups of each 3-bpp ligand is deprotonated. The deprotonation of the N-heterocyclic ligand and the simultaneous oxidation of the metal ion, as in a PCET-based process, was earlier observed in iron(II) complexes of 2,6-diimidazolyl pyridines [51]. In contrast, the deprotonation of only one OH group of 6,6′-dihydroxy terpyridine in its copper(II) complexes retained the oxidation state of the metal ion [55], as in [Fe(L^O−^)_2_]•5CH_3_OH.

In [Fe(L^OH^)_2_][FeCl_4_]•5CH_3_CN, which features the anion FeCl_4_^−^ resulting from the coordinative nature of the chloride anion, the Fe-N bond lengths and continuous shape measures [56] (Table 1) fall into the range typical of low-spin complexes of iron(II) with 3-bpp ligands [21]. One OH group of each 3-bpp ligand in [Fe(L^OH^)_2_][FeCl_4_]•5CH_3_CN forms hydrogen bonds O-H…Cl (O…Cl 3.057(8) and 3.050(6) Å, OHCl 155.3(5) and 166.6(4)°) with the anions FeCl_4_^2−^ to produce hydrogen-bonded spirals along the crystallographic axis *b* (Figure 1, middle). The other two OH groups are hydrogen-bonded to the lattice molecules of acetonitrile (O…N 2.860(16) and 2.721(12) Å, OHN 132.1(6) and 168.9(5)°); other acetonitrile molecules occur inside and between the above spirals.

The coordination of the iron(II) ion by the p-cyanophenyl group of the ligand does not occur, probably owing to the kinetics of the reaction, which causes the complex [Fe(L^OH^)_2_][FeCl_4_]•5CH_3_CN to precipitate from the reaction mixture faster than the iron(II) ion coordinates.

With CuSO_4_ 6H_2_O in methanol, the complex [Fe(L^OH^)_2_](BF_4_)_2_ also does not act as a linker to the copper(II) ion. Instead, it transforms into a neutral iron(II) complex [Fe(L^O−^)_2_]•5CH_3_OH (Figure 2, top), with one of the two OH groups in each 3-bpp ligand being deprotonated. Under these conditions, the deprotonation may occur through a multi-site PCET process with an electron transfer from the solvent (methanol to produce formaldehyde) rather than from the iron(II) ion, which keeps its oxidation state 2+, similar to copper complexes of 6,6′-dihydroxy terpyridine [55]. The deprotonation of the OH groups causes the shortening of the bonds Fe-N (Table 1) with the pyrazol-3-yl moieties while those with the pyridine moiety remain virtually the same (av. 1.978 and 2.002 Å vs. 1.916 and 1.916 Å in [Fe(L^O−^)_2_]•5CH_3_OH and [Fe(L^OH^)_2_][FeCl_4_]•5CH_3_CN, respectively), thereby confirming the low-spin state of the iron(II) ion. The remaining OH group of one the 3-bpp ligand forms charge-assisted hydrogen bonds O-H…O-(O…O 2.467(6) Å, OHO 172.7(3)°) with its deprotonated counterpart from the second ligand of the complex [Fe(L^O−^)_2_] to produce hydrogen-bonded zig-zag chains along the crystallographic axis *b* (Figure 1, bottom), which are additionally stabilized by hydrogen bonds with the solvate methanol molecules (O…O 2.549(13)–2.904(7) Å, OHO 150.8(6)–172.3(6)°). Such a strong hydrogen bonding is, apparently, responsible for the insolubility of the complex [Fe(L^O−^)_2_]•5CH_3_OH in methanol, so it precipitates faster than the copper ion coordinates the p-cyanophenyl group of the ligand in the reaction mixture.

Under the same mild conditions of reactant diffusion, the reaction of [Fe(L^OH^)_2_](BF_4_)_2_ with AgNO_3_ in methanol resulted in a 1D-CP [Fe(L^O−^)2AgNO_3_BF_4_•CH_3_OH]n•1.75nCH_3_OH•nH_2_O, which is the second homoleptic CP with a 3-bpp ligand [32] and the first one to retain a core N_3_(L)MN_3_(L) (Figure 2, top). As follows from X-ray diffraction, the Fe-N bond lengths (Table 1) fall into the range typical of (pseudo)octahedral complexes of iron(III) in the low-spin state [21]. Three cores [Fe(L^O−^)_2_] coordinate the silver(I) ion via the p-cyanophenyl substituent of the 3-bpp ligand and one of the two deprotonated OH groups (Ag-N 2.147(7) and 2.178(7) Å, Ag-O 2.498(7) Å) to produce a coordination double chain along the diagonal of the crystallographic plane *a0c* (Figure 2, bottom). The seesaw-shaped coordination environment, as gauged by continuous symmetry measures [56], of the silver(I) ion is completed by the oxygen atom of the coordinated methanol molecule (Ag-O 2.448(7) Å). The nitrate and tetrafluoroborate anions occur in the outer sphere together with solvent methanol and water molecules that are hold together by O-H…O hydrogen bonds with each other (O…O 2.591(19)–3.14(2) Å, OHO 128(14)–173.1(7)°; O…F 2.785(12) Å, OHF 154.9(5)°) and with the coordinated methanol molecule (O…O 2.724(12) Å, OHO 120.7(5)°). The above chains are packed by the charge-assisted hydrogen bonds (O…O 2.447(8) and 2.481(8) Å, OHO 159.6(5) and 165.5(5)°) between the second deprotonated OH group and its two non-deprotonated analogues in the crystallographic plane *a0b*. Weak parallel-displaced stacking interactions additionally occur between the dichlorophenyl and the p-cyanophenyl groups of neighboring cores [Fe(L^O−^)_2_].

Upon the precipitation of CP 1D-CP [Fe(L^O−^)2AgNO_3_BF_4_•CH_3_OH]n•1.75nCH_3_OH•nH_2_O, a complete discoloration of the solution of [Fe(L^OH^)_2_](BF_4_)_2_ was observed, thereby indicating that no starting material was left in a solution. Under these conditions, the deprotonation of one of the OH groups was not expected in contrast to the oxidation of iron(II) to iron(III) [57]. In the absence of a strong base, the former may arise from a PCET [46] with both the electron and proton transferred towards the anion NO_3_^−^ similar to iron(II) complexes with imidazole-based ligands [51]. The resulting iron(III)-containing CP is not stable towards DMF, which causes its decomposition to produce a dark brown solution featuring signals of unidentified diamagnetic compounds in its ^1^H NMR spectra.

Mild conditions of reagent diffusion allowed us to keep the core N_3_(L)MN_3_(L) of the parent complex [Fe(L^OH^)_2_](BF_4_)_2_ intact with no signs of ligand dissociation or metal metathesis. However, the reactions with FeCl_2_ and CuSO_4_ did not produce a CP, possibly due to the fast precipitation of the side products [Fe(L^OH^)_2_][FeCl_4_]•5CH_3_CN and [Fe(L^O−^)_2_]•5CH_3_OH and lower rate of copper coordination. Harsher conditions of the solvothermal synthesis might help overcoming this problem.

**Figure 3:**
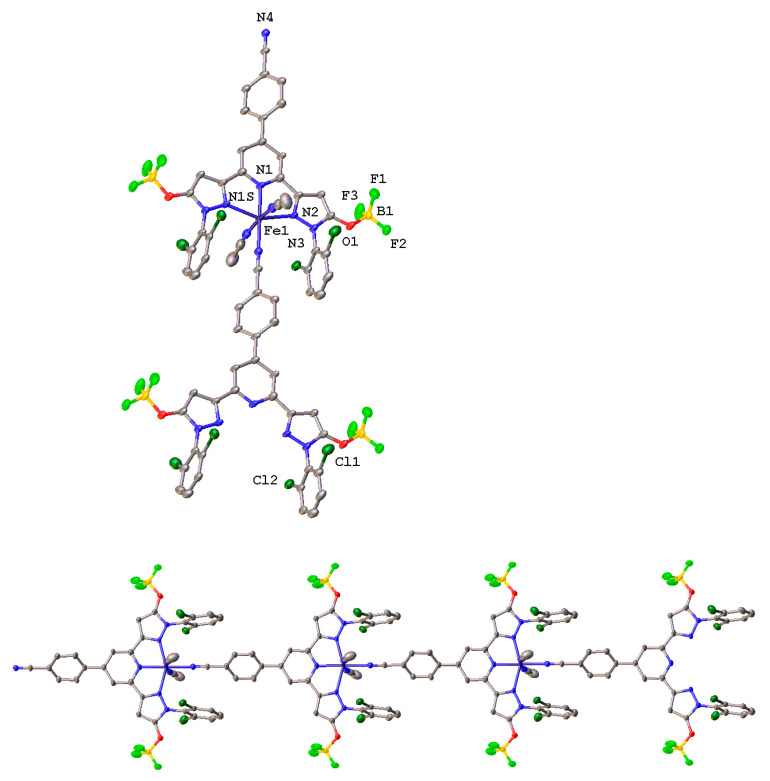
A general view of the complex species [Fe(L^OBF^_3_)_2_(CH_3_CN)_2_] (**top**) and a fragment of the 1D-coordination polymer (**bottom**) in [Fe(L^OBF3^)(CH_3_COO)(CH_3_CN)_2_]_n_•nCH_3_CN. Acetate anions, solvent acetonitrile molecules and hydrogen atoms are omitted, non-hydrogen atoms are shown as thermal ellipsoids (*p* = 30%) and only labels of the heteroatoms in an asymmetric part of the unit cell are given; the ligand and the metal ion occupy the special positions, the two-fold axis.

Under solvothermal conditions often used to obtain new CPs and MOFs [25,53], the complex [Fe(L^OH^)_2_](BF_4_)_2_ was kept at 140 °C for 24 h in a sealed ampule with a solution of the transition metal salt in DMF or DMF/AcN (1:1). In most cases, no crystalline products were obtained; ^1^H NMR spectroscopy of the reaction mixtures revealed a variety of paramagnetic compounds as a sign of the decomposition of the parent complex [Fe(L^OH^)_2_](BF_4_)_2_. The only exception was cobalt acetate tetrahydrate, which produced the third homoleptic CP with a 3-bpp ligand [32], a 1D-CP of iron(III) [Fe(L^OBF^_3_)(CH_3_COO)(CH_3_CN)_2_]n•nCH_3_CN with the OH groups transformed into OBF_3_ upon the reaction with BF_4_ (Figure 3, top). The pseudo-octahedral coordination environment of the metal ion, as gauged by continuous symmetry measures [56], is formed by the three nitrogen atoms of the tridentate 3-bpp ligand, the nitrogen atom of the p-cyanophenyl substituent of the other 3-bpp ligand and two nitrogen atoms of the solvent acetonitrile molecules; the Fe-N bond lengths (Table 1) fall into the range typical of low-spin complexes of iron(III) [21]. The 3-bpp ligand acts as a bridge to produce 1D-coordination polymer chains along the crystallographic axis *b* (Figure 3, bottom). Parallel-displaced stacking interactions between the parallel dichlorophenyl groups of the neighboring chains that pack them along the crystallographic axis *c* into the layers; the appropriate intercentroid and shift distances are 3.933(7) and 1.956(15) Å. Acetate anions and solvent acetonitrile molecules occur between these layers.

The 1D-CP [Fe(L^OBF3^)(CH_3_COO)(CH_3_CN)_2_]_n_•nCH_3_CN apparently resulted from the heat-induced dissociation [58] of the complex [Fe(L^OH^)_2_](BF_4_)_2_ and further coordination of the “open-shell” iron(II) ion by the p-cyanophenyl group of the 3-bpp ligand in acetonitrile. Long-term heating also causes other reactions to occur, such as OH bond activation [59]. At this temperature, the PCET process involves the tetrafluoroborate anion, as it is the only potential source of BF_3_ moiety. The hydrogen atom transfer via a PCET towards the anion BF_4_ apparently produces HF and BF_3_ (Lewis acid) and the latter reacts with the deprotonated ligand (Lewis base), thereby resulting in the formation of the CP [Fe(L^OBF3^)(CH_3_COO)(CH_3_CN)_2_]_n_•nCH_3_CN (see Figure S1 of Supplementary Materials). Here, cobalt acetate acts only as a donor of the acetate anion rather than a source of the metal ion to coordinate the p-cyanophenyl group, owing to higher stability of the iron(II)-cyano species compared to those of cobalt(II) [60]. In such a multicomponent system of cobalt acetate, [Fe(L^OH^)_2_](BF_4_)_2_ and DMF/AcN, however, it is quite difficult to pinpoint the exact mechanism of the PCET-assisted transformation. The reason behind it may be high temperature or the presence of oxygen, but it is still arguable.

## 3. Materials and Methods

Synthesis. All synthetic manipulations were carried on air unless stated otherwise. Solvents were purchased from commercial sources and purified by distilling from conventional drying agents in an argon atmosphere prior to use. The iron(II) complex [Fe(L^OH^)_2_](BF_4_)_2_ was synthesized as reported previously [44].

[Fe(L^OH^)_2_][FeCl_4_]•5CH_3_CN. In a 3 mL vial, [Fe(L^OH^)_2_](BF_4_)_2_ (30 mg, 0.0225 mmol) was dissolved in 5 mL of acetonitrile and FeCl_2_ (6 mg, 0.047 mmol) was added. The resulting solution was stirred for 30 min, layered with Et_2_O and kept overnight to produce red crystals of the product. These were centrifuged, washed with methanol and dried in vacuum. Yield: 27 mg (70%). Anal. Calc. for C_60_H_32_Cl_12_Fe_2_N_12_O_4_∙5AcN (%): C, 48.67; H, 2.74; N, 13.79. Found (%): C, 49.78; H, 2.89; N, 13.58. The disordered molecule of diethyl ether found in the crystals by X-ray diffraction (see Section 2) was, apparently, lost upon their drying.

[Fe(L^O−^)_2_]•5CH_3_OH. In a 3 mL vial, [Fe(L^OH^)_2_](BF_4_)_2_ (60 mg, 0.045 mmol) was dissolved in dissolved in 3 mL of methanol. Another 3 mL vial was charged with CuSO_4_•5H_2_O (56 mg, 0.225 mmol) dissolved in 3 mL of methanol. The two vials were placed into another 20 mL vial (see Figure S2 of Supplementary Materials), which was filled with methanol above the level of these vials and kept for two days until all the reagents diffused into the 20 mL vial and red crystals of the product appeared. The resulting crystals were centrifuged, washed with methanol and dried in vacuum. Yield: 31 mg (46%). Anal. Calc. for C_60_H_30_Cl_8_FeN_12_O_4_∙5CH_3_OH (%): C, 52.66; H, 3.40; N, 11.34; Found (%): C, 52.50; H, 3.50; N, 11.22. The disordered molecule of methanol found in the crystals by X-ray diffraction (see Section 2) was, apparently, lost upon their drying.

[Fe(L^O−^)_2_AgNO_3_BF_4_•CH_3_OH]n•1.75nCH_3_OH•nH_2_O. In a 3 mL vial, [Fe(L^OH^)_2_](BF_4_)_2_ (60 mg, 0.045 mmol) was dissolved in 3 mL of methanol. Another 3 mL vial was charged with AgNO_3_ (77 mg, 0.45 mmol) dissolved in 3 mL of methanol. The two vials were placed into another 20 mL vial (see Figure S2 of Supplementary Materials), which was filled with methanol above the level of these vials and kept for two days until all the reagents diffused into the 20 mL vial and grey crystals of the product appeared. These were centrifuged, washed with methanol and dried in vacuum. Yield: 43 mg (56%). Anal. Calc. for C_62.75_H_43_AgBCl_8_F_4_FeN_13_O_10.75_ (%): C, 44.72; H, 2.57; N, 10.81. Found (%): C, 44.86; H, 2.37; N, 10.01.

[Fe(L^OBF^_3_)(CH_3_COO)(CH_3_CN)_2_]_n_•nCH_3_CN. A mixture of [Fe(L^OH^)_2_](BF_4_)_2_ (20 mg, 0.015 mmol) and Co(OAc)_2_•4H_2_O (6 mg, 0.1 mmol) was dissolved in 1 mL of a 1:1 mixture of acetonitrile and N,N’-dimethyl formamide and stirred at room temperature for 15 min. The reaction mixture was transferred to a 1 mL ampule that was sealed and then heated at 140 °C for 24 h. The resulting mixture was cooled to room temperature at a rate of 5 °C/min to produce yellow crystals. Yield: 12 mg (40%). Anal. Calc. for C_76_H_52_B_4_Cl_8_F_12_Fe_2_N_18_O_8_ (%): C, 45.37; H, 2.61; N, 12.53. Found (%): C, 45.43; H, 2.44; N, 12.23.

X-ray crystallography. X-ray diffraction data for [Fe(L^OBF^_3_)(CH_3_COO)(CH_3_CN)_2_]_n_•nCH_3_CN were collected at 100 K at the protein station of Kurchatov Centre for Synchrotron radiation (λ = 0.745 Å), and those for all others at 120 K with a Bruker APEX2 DUO CCD diffractometer, using the graphite monochromated Mo-Kα radiation (λ = 0.71073 Å). Using Olex [2,61], the structures were solved with the ShelXT structure solution program [62] using Intrinsic Phasing and refined with the XL refinement package [63] using Least-Squares minimization. Hydrogen atoms of OH groups in [Fe(L^O−^)_2_AgNO_3_BF_4_•CH_3_OH]n•1.75nCH_3_OH•nH_2_O, [Fe(L^OH^)_2_][FeCl_4_]•5CH_3_CN and [Fe(L^O−^)_2_]•5CH_3_OH, and of water and methanol molecules in [Fe(L^O−^)_2_AgNO_3_BF_4_•CH_3_OH]n•1.75nCH_3_OH•nH_2_O and [Fe(L^O−^)_2_]•5CH_3_OH, were located in difference Fourier synthesis, while positions of other hydrogen atoms were calculated, and they all were refined in the isotropic approximation in the riding model. The unit cells of [Fe(L^OH^)_2_][FeCl_4_]•5CH_3_CN and [Fe(L^O−^)_2_]•5CH_3_OH contain additional solvate molecule of diethyl ether or methanol, respectively, which was severely disordered and thereby treated as a diffuse contribution to the overall scattering without specific atom positions by SQUEEZE/PLATON [64]. Crystal data and structure refinement parameters are given in Table 2. CCDC 2245235, 2245236, 2245234 and 2245233 contain the supplementary crystallographic data for [Fe(L^OH^)_2_][FeCl_4_]•5CH_3_CN, [Fe(L^O−^)_2_]•5CH_3_OH, [Fe(L^O−^)_2_AgNO_3_BF_4_•CH_3_OH]n•1.75nCH_3_OH•nH_2_O and [Fe(L^OBF^_3_)(CH_3_COO)(CH_3_CN)_2_]_n_•nCH_3_CN, respectively.

## 4. Conclusions

Under mild conditions of the reactant diffusion and harsh solvothermal conditions, we synthesized—from the single complex [Fe(L^OH^)_2_](BF_4_)_2_—new 1D-coordination polymers of iron(III) [Fe(L^O−^)_2_AgNO_3_BF_4_•CH_3_OH]n•1.75nCH_3_OH•nH_2_O and [Fe(L^OBF^_3_)(CH_3_COO)(CH_3_CN)_2_]_n_•nCH_3_CN; they are the second and the third homoleptic CPs to contain the 3-bpp ligand and the heterometallic one is the first to retain the core N_3_(L)MN_3_(L). These coordination polymers were obtained by applying a novel approach that includes a PCET from the hydroxyl group of the pyrazolone moiety to the iron(II) ion. The deprotonated 3-bpp scaffold, which appeared in the neutral complex [Fe(L^O−^)_2_]•5CH_3_OH via a potential multi-site PCET process similar to copper complexes with 6,6’-dihydroxy terpyridine, stabilized the low-spin state of the metal ion—as gauged by X-ray diffraction—owing to its anionic character. The proposed approach can be applied to other ligands with acidic protons, such as substituted pyrazolones [65,66], and other metal ions that are prone to oxidation to an SCO-active form, such as manganese(II) and cobalt(II) [67], to produce switchable CPs and MOFs.

Under harsh solvothermal conditions, retaining the core N_3_(L)MN_3_(L) with a neutral ligand, such as 1- or 3-bpp and tpy, is hardly possible. The heating initiated a PCET between the complex [Fe(L^OH^)_2_](BF_4_)_2_ and the tetrafluoroborate anion, which is believed to be inert towards reactive species, such as cation radicals [68]. Our investigation of this PCET reaction as a catalytic variant of the hydrogen atom transfer for a reductive ketone coupling [69] is currently underway.

## Figures and Tables

**Table 1:** Main geometric parameters and continuous symmetry measures ^[a]^ as obtained from X-ray diffraction for [Fe(L^OH^)_2_][FeCl_4_]•5CH_3_CN, [Fe(L^O−^)_2_]•5CH_3_OH, [Fe(L^O−^)_2_AgNO_3_BF_4_•CH_3_OH]n•1.75nCH_3_OH•nH_2_O and [Fe(L^OBF^_3_)(CH_3_COO)(CH_3_CN)_2_]n•nCH_3_CN.

Parameter	[Fe(L^OH^)_2_] [FeCl_4_]•5CH_3_CN	[Fe(L^O−^)_2_]• 5CH_3_OH	[Fe(L^O−^)_2_ AgNO_3_BF_4_•CH_3_OH]_n_•1.75nCH_3_OH•nH_2_O	[Fe(L^OBF^_3_)(CH_3_COO)(CH_3_CN)_2_]_n_• nCH_3_CN
Fe-N_Py_, Å	1.920(6), 1.912(6)	1.918(5), 1.914(5)	1.915(6), 1.904(6)	2.061(8)
Fe-N_Pz_, Å	1.999(5)–2.005(6)	1.967(5)–1.998(5)	1.914(7)–1.959(7)	2.145(6)
Fe-N_CN_, Å	-	-	-	2.079(8) [2.117(10)] ^[b]^
θ, °	88.44(6)	89.34(6)	88.67(7)	0.0
ϕ, °	179.5(3)	179.6(3)	178.7(3)	179.4(5) ^[c]^
β, °	38.3(3), 36.0(3)	21.4(2), 40.5(3)	29.1(3), 39.9(4) [23.2(14)] ^[d]^	48.9(5)
γ, °	85.3(3)–89.3(3)	82.5(3)–89.7(3)	85.3(3)–88.2(3)	82.8(4)
S(OC)	2.486	2.280	1.895	2.011
S(TPR)	12.088	12.373	12.548	12.844
S(ebcT)	12.824	12.936	13.386	14.110
S(SS)	-	-	2.426	-
S(T)	-	-	4.975	

^[a]^ θ is the ‘twist’ angle between the two least-squares planes of the 3-bpp ligands; ϕ is the ‘rotation’ angle N_Py_-Fe-N_Py_; β is the rotation angle of the p-cyanophenyl group relative to the pyridine plane; γ is the rotation angle of the dichlorophenyl group relative to the pyrazol-3-yl plane; S(OC), S(TPR) and S(ebcT) are octahedral, trigonal-prismatic and edge-bicapped tetrahedral symmetry measures, respectively; S(SS) and S(T) are seesaw and tetrahedral symmetry measures, respectively. ^[b]^ The value in brackets is for the coordination bond with the acetonitrile molecule. ^[c]^ The value for the angle N_Py_-Fe-N_CN_ is given. ^[d]^ The value in brackets is for the minor component of the disordered p-cyanophenyl group.

**Table 2:** Crystal data and structure refinement parameters for [Fe(L^OH^)_2_][FeCl_4_]•5CH_3_CN, [Fe(L^O−^)_2_]•5CH_3_OH, [Fe(L^O−^)_2_AgNO_3_BF_4_•CH_3_OH]n•1.75nCH_3_OH•nH_2_O and [Fe(L^OBF^_3_)(CH_3_COO)(CH_3_CN)_2_]_n_•nCH_3_CN.

	[Fe(L^OH^)_2_][FeCl_4_]•5CH_3_CN	[Fe(L^O−^)_2_]•5CH_3_OH	[Fe(L^O−^)_2_AgNO_3_BF_4_• CH_3_OH]_n_•1.75nCH_3_OH•nH_2_O	[Fe(L^OBF^_3_)(CH_3_COO) (CH_3_CN)_2_]_n_• nCH_3_CN
Formula unit	C_70_H_47_Cl_12_Fe_2_ N_17_O_4_	C_65_H_50_Cl_8_FeN_12_O_9_	C_62.75_H_43_AgBCl_8_F_4_FeN_13_O_10.75_	C_76_H_52_B_4_Cl_8_F_12_ Fe_2_N_18_O_8_
Formula weight	1727.34	1482.62	1685.23	2011.89
Temperature, K	120	120	120	100
Crystal system	Monoclinic	Monoclinic	Monoclinic	Monoclinic
Space group	P2_1_/n	P2_1_/c	P2_1_/c	C2/c
Z	4	4	4	2
a, Å	19.095(4)	20.951(4)	15.792(5)	17.488(4)
b, Å	18.899(4)	14.806(3)	16.059(5)	13.740(3)
c, Å	22.565(5)	23.122(4)	26.900(8)	21.498(4)
α, °	90	90	90	90
β, °	91.163(4)	110.525(4)	106.675(6)	104.66(3)
γ	90	90	90	90
V, Å^3^	8141(3)	6717(2)	6535(3)	4997.6(18)
Dcalc (g cm^−1^)	1.409	1.466	1.713	1.337
Linear absorption, μ (cm^−1^)	8.06	6.09	9.33	6.59
F(000)	3496	3032	3386	2028
2Θmax, °	52	52	54	52.6
Reflections measured	103,671	80,169	90,879	12,966
Independent reflections	15,995	13,197	14,264	4418
Observed reflections [I > 2σ(I)]	8258	6414	5673	2302
Parameters	955	861	940	328
R1	0.0830	0.0729	0.0742	0.0944
wR2	0.2441	0.2152	0.2071	0.3228
GOF	1.041	0.976	0.963	1.034
Δρ_max_/Δρ_min_ (e Å^−3^)	1.539/−0.750	1.191/−0.879	1.064/−0.840	0.998/−0.527

## Data Availability

The X-ray diffraction data are available from Cambridge Structural Database under CCDC numbers 2245235, 2245236, 2245234 and 2245233.

## Supplementary Materials

The following supporting information can be downloaded at: https://www.mdpi.com/article/10.3390/molecules28114275/s1, Figure S1: Plausible mechanism for the PCET-assisted formation of [Fe(LOBF3)(CH3COO)(CH3CN)2]n•nCH3CN; Figure S2: Reactant diffusion technique used for crystal growth.

## References

[1] Freund R., Zaremba O., Arnauts G., Ameloot R., Skorupskii G., Dincă M., Bavykina A., Gascon J., Ejsmont A., Goscianska J. (2021). The Current Status of MOF and COF Applications. Angew. Chem. Int. Ed..

[2] Cheng W., Tang X., Zhang Y., Wu D., Yang W. (2021). Applications of Metal-Organic Framework (MOF)-Based Sensors for Food Safety: Enhancing Mechanisms and Recent Advances. Trends Food Sci. Technol..

[3] Ali M., Pervaiz E., Noor T., Rabi O., Zahra R., Yang M. (2021). Recent advancements in MOF-based catalysts for applications in electrochemical and photoelectrochemical water splitting: A review-Ali-2021. Int. J. Energy Res.-Wiley Online Libr..

[4] Qian H.Y. (2017). Synthesis Characterization, Crystal Structures, and Antibacterial Activity of 8-Hydroxyquinoline-Coordinated Oxidovanadium(V) Complexes with Tridentate Hydrazone Ligands. Russ. J. Coord. Chem..

[5] Castellanos S., Kapteijn F., Gascon J. (2016). Photoswitchable Metal Organic Frameworks: Turn on the Lights and Close the Windows. CrystEngComm.

[6] Manrique-Juárez M.D., Rat S., Salmon L., Molnár G., Quintero C.M., Nicu L., Shepherd H.J., Bousseksou A. (2016). Switchable Molecule-Based Materials for Micro- and Nanoscale Actuating Applications: Achievements and Prospects. Coord. Chem. Rev..

[7] Bigdeli F., Lollar C.T., Morsali A., Zhou H.-C. (2020). Switching in Metal–Organic Frameworks. Angew. Chem. Int. Ed..

[8] Brown J.W., Henderson B.L., Kiesz M.D., Whalley A.C., Morris W., Grunder S., Deng H., Furukawa H., Zink J.I., Stoddart J.F. (2013). Photophysical Pore Control in an Azobenzene-Containing Metal–Organic Framework. Chem. Sci..

[9] Müller K., Knebel A., Zhao F., Bléger D., Caro J., Heinke L. (2017). Switching Thin Films of Azobenzene-Containing Metal–Organic Frameworks with Visible Light. Chem.– Eur. J..

[10] Park J., Feng D., Yuan S., Zhou H.-C. (2015). Photochromic Metal–Organic Frameworks: Reversible Control of Singlet Oxygen Generation. Angew. Chem..

[11] Luo F., Fan C.B., Luo M.B., Wu X.L., Zhu Y., Pu S.Z., Xu W.-Y., Guo G.-C. (2014). Photoswitching CO2 Capture and Release in a Photochromic Diarylethene Metal–Organic Framework. Angew. Chem..

[12] Nagata S., Kokado K., Sada K. (2015). Metal–Organic Framework Tethering PNIPAM for ON–OFF Controlled Release in Solution. Chem. Commun..

[13] Sun J.-K., Cai L.-X., Chen Y.-J., Li Z.-H., Zhang J. (2011). Reversible Luminescence Switch in a Photochromic Metal–Organic Framework. Chem. Commun..

[14] Ogihara N., Ohba N., Kishida Y. (2017). On/off switchable electronic conduction in intercalated metal-organic frameworks. Sci. Adv..

[15] Niel V., Thompson A.L., Muñoz M.C., Galet A., Goeta A.E., Real J.A. (2003). Crystalline-State Reaction with Allosteric Effect in Spin-Crossover, Interpenetrated Networks with Magnetic and Optical Bistability. Angew. Chem. Int. Ed..

[16] Liu F.-L., Li D., Su L.-J., Tao J. (2018). Reversible Three Equal-Step Spin Crossover in an Iron(II) Hofmann-Type Metal–Organic Framework. Dalton Trans..

[17] Halcrow M.A. (2016). The Effect of Ligand Design on Metal Ion Spin State—Lessons from Spin Crossover Complexes. Crystals.

[18] Ohkoshi S., Imoto K., Tsunobuchi Y., Takano S., Tokoro H. (2011). Light-induced spin-crossover magnet|Nature Chemistry. Nat. Chem..

[19] Tarafder K., Kanungo S., Oppeneer P.M., Saha-Dasgupta T. (2012). Pressure and Temperature Control of Spin-Switchable Metal-Organic Coordination Polymers from Ab Initio Calculations. Phys. Rev. Lett..

[20] Gong L.L., Feng X.F., Luo F., Yi X.F., Zheng A.M. (2016). Removal and Safe Reuse of Highly Toxic Allyl Alcohol Using a Highly Selective Photo-Sensitive Metal–Organic Framework. Green Chem..

[21] Halcrow M.A. (2013). Spin-Crossover Materials: Properties and Applications.

[22] Southon P.D., Liu L., Fellows E.A., Price D.J., Halder G.J., Chapman K.W., Moubaraki B., Murray K.S., Létard J.-F., Kepert C.J. (2009). Dynamic Interplay between Spin-Crossover and Host−Guest Function in a Nanoporous Metal−Organic Framework Material. J. Am. Chem. Soc..

[23] Bao X., Liu J.-L., Leng J.-D., Lin Z., Tong M.-L., Nihei M., Oshio H. (2010). Spin Crossover versus Low-Spin Behaviour Exhibited in 2D and 3D Supramolecular Isomers of [FeII(2,4-Bpt)2]⋅Guest. Chem.– Eur. J..

[24] Halcrow M.A. (2011). Structure:Function Relationships in Molecular Spin-Crossover Complexes. Chem. Soc. Rev..

[25] Stock N., Biswas S. (2012). Synthesis of Metal-Organic Frameworks (MOFs): Routes to Various MOF Topologies, Morphologies, and Composites. Chem. Rev..

[26] Nikovskiy I., Aleshin D.Y., Novikov V.V., Polezhaev A.V., Khakina E.A., Melnikova E.K., Nelyubina Y.V. (2022). Selective Pathway toward Heteroleptic Spin-Crossover Iron(II) Complexes with Pyridine-Based *N* -Donor Ligands. Inorg. Chem..

[27] Bommakanti S., Venkataramudu U., Das S.K. (2019). Functional Coordination Polymers from a Bifunctional Ligand: A Quantitative Transmetalation via Single Crystal to Single Crystal Transformation. Cryst. Growth Des..

[28] Berdiell I.C., Warriner S.L., Halcrow M.A. (2018). Silver (I) complexes of bis-and tris-(pyrazolyl) azine derivatives–dimers, coordination polymers and a pentametallic assembly. Dalton Trans..

[29] Li L.-L., Liu L.-L., Ren Z.-G., Li H.-X., Zhang Y., Lang J.-P. (2009). Solvothermal Assembly of a Mixed-Valence Cu(I,II) Cyanide Coordination Polymer [Cu(II)Cu(I)2(µ-Br)2(µ-CN)2(Bdmpp)]n by C–C Bond Cleavage of Acetonitrile. CrystEngComm.

[30] Liu G.-F., Ren Z.-G., Li H.-X., Chen Y., Li Q.-H., Zhang Y., Lang J.-P. (2007). Homo- and Heterometallic Coordination Oligomers and Polymers Derived from the Preformed Complexes [Cu(Bdmpp)(MeCN)2](ClO4)2, [Cu(Bdmpp)(N3)2], and [Cu(Bdmpp)(N3)(μ-N3)]2 [Bdmpp = 2,6-Bis(3,5-Dimethyl-1H-Pyrazol-1-Yl)Pyridine]: Syntheses, Structures, and Redox Properties. Eur. J. Inorg. Chem..

[31] Lazarou K.N., Chadjistamatis I., Terzis A., Perlepes S.P., Raptopoulou C.P. (2010). Complexes Derived from the Copper(II)/Succinamic Acid/N,N′,N″-Chelate Tertiary Reaction Systems: Synthesis, Structural and Spectroscopic Studies. Polyhedron.

[32] Wang X., Bai F.Y., Xing Y.H., Wan L.J., Guan Q.L., Hou Y.N., Zhang R. (2014). Experimental, Characteristic Evidence and Surface Photovoltage Properties of a Series of Cu/Mn Complexes with Bipyrazolyl-Pyridine and Different Spanning Dicarboxylate. Inorg. Chim. Acta.

[33] Zhang X., Xing N., Bai F., Wan L., Shan H., Hou Y., Xing Y., Shi Z. (2013). Multi-Functional D10 Metal–Organic Materials Based on Bis-Pyrazole/Pyridine Ligands Supported by a 2,6-Di(3-Pyrazolyl)Pyridine with Different Spanning Flexible Dicarboxylate Ligands: Synthesis, Structure, Photoluminescent and Catalytic Properties. CrystEngComm.

[34] Elahi S.M., Raizada M., Sahu P.K., Konar S. (2021). Terpyridine-Based 3D Metal–Organic-Frameworks: A Structure–Property Correlation. Chem.– Eur. J..

[35] Zhilina E.F., Chizhov D.L., Sidorov A.A., Aleksandrov G.G., Kiskin M., Slepukhin P.A., Fedin M., Starichenko D.V., Korolev A.V., Shvachko Y.N. (2013). Neutral Tetranuclear Cu(II) Complex of 2,6-Di(5-Trifluoromethylpyrazol-3-Yl)Pyridine: Synthesis, Characterization and Its Transformation with Selected Aza-Ligands. Polyhedron.

[36] Yang Z.N., Sun T.T. (2008). Catena-Poly[[[2,6-Bis-(Pyrazol-1-Yl-ΚN)Pyridine-ΚN](Nitrato-ΚO,O’)Cadmium(II)]-μ-Thio-Cyanato-ΚN:S]. Acta Cryst. Sect E Struct Rep. Online.

[37] Wu Q., Xing N., Liu X., Xu L., Ma X., Yan Z., Xing Y. (2015). Two Novel Cu(II) Complexes: Synthesis, Structure and Application in C–H Bond Activation. Polyhedron.

[38] Cui H.-L., Zhan S.-Z., Li M., Ng S.W., Li D. (2011). Luminescent Isomeric Pr–Ag Coordination Polymers Immobilized with Organic Sensitizer and Ag–S Clusters. Dalton Trans..

[39] Kang X.M., Wang W.M., Yao L.H., Ren H.X., Zhao B. (2018). Solvent-dependent variations of both structure and catalytic performance in three manganese coordination polymers. Dalton Trans..

[40] Zhou Y., Chen W., Wang D. (2008). Mononuclear, Dinuclear, Hexanuclear, and One-Dimensional Polymeric Silver Complexes Having Ligand-Supported and Unsupported Argentophilic Interactions Stabilized by Pincer-like 2,6-Bis(5-Pyrazolyl)Pyridine Ligands. Dalton Trans..

[41] Cook B.J., Pink M., Chen C.-H., Caulton K.G. (2018). Electrophile Recruitment as a Structural Element in Bis-Pyrazolate Pyridine Complex Aggregation. Eur. J. Inorg. Chem..

[42] Cook B.J., Polezhaev A.V., Chen C.-H., Pink M., Caulton K.G. (2017). Deprotonation, Chloride Abstraction, and Dehydrohalogenation as Synthetic Routes to Bis-Pyrazolate Pyridyl Iron (II) Complexes. Eur. J. Inorg. Chem..

[43] Nikovskiy I., Polezhaev A.V., Novikov V.V., Aleshin D., Pavlov A.A., Saffiulina E., Aysin R.R., Dorovatovskii P., Nodaraki L., Tuna F. (2020). Towards Molecular Design of Spin-Crossover Complexes of 2,6-Bis(Pyrazol-3-Yl)Pyridines. Chem. Eur. J..

[44] Aleshin D.Y., Nikovskiy I., Novikov V.V., Polezhaev A.V., Melnikova E.K., Nelyubina Y.V. (2021). Room-Temperature Spin Crossover in a Solution of Iron(II) Complexes with N,N′-Disubstituted Bis(Pyrazol-3-Yl)Pyridines. ACS Omega.

[45] Weinberg D.R., Gagliardi C.J., Hull J.F., Murphy C.F., Kent C.A., Westlake B.C., Paul A., Ess D.H., McCafferty D.G., Meyer T.J. (2012). Proton-Coupled Electron Transfer. Chem. Rev..

[46] Tyburski R., Liu T., Glover S.D., Hammarström L. (2021). Proton-Coupled Electron Transfer Guidelines, Fair and Square. J. Am. Chem. Soc..

[47] Gentry E.C., Knowles R.R. (2016). Synthetic Applications of Proton-Coupled Electron Transfer. Acc. Chem. Res..

[48] Miller D.C., Tarantino K.T., Knowles R.R. (2016). Proton-Coupled Electron Transfer in Organic Synthesis: Fundamentals, Applications, and Opportunities. Top. Curr. Chem. (Z).

[49] Yang J.-D., Ji P., Xue X.-S., Cheng J.-P. (2018). Recent Advances and Advisable Applications of Bond Energetics in Organic Chemistry. J. Am. Chem. Soc..

[50] Rono L.J., Yayla H.G., Wang D.Y., Armstrong M.F., Knowles R.R. (2013). Enantioselective Photoredox Catalysis Enabled by Proton-Coupled Electron Transfer: Development of an Asymmetric Aza-Pinacol Cyclization. J. Am. Chem. Soc..

[51] Brewer C., Brewer G., Luckett C., Marbury G.S., Viragh C., Beatty A.M., Scheidt W.R. (2004). Proton Control of Oxidation and Spin State in a Series of Iron Tripodal Imidazole Complexes. Inorg. Chem..

[52] Kirov G.K., Chernov A.A. (1984). Theory of Diffusion Methods of Growing Crystals. Рoст Кристаллoь/Rost Kristallov/Growth of Crystals.

[53] Zhao Y., Li K., Li J. (2010). Solvothermal Synthesis of Multifunctional Coordination Polymers. Z. Für Nat. B.

[54] Luz I., Toy L., Rabie F., Lail M., Soukri M. (2019). Synthesis of Soluble Metal Organic Framework Composites for Mixed Matrix Membranes. ACS Appl. Mater. Interfaces.

[55] Dahl E.W., Szymczak N.K. (2016). Hydrogen Bonds Dictate the Coordination Geometry of Copper: Characterization of a Square-Planar Copper(I) Complex. Angew. Chem. Int. Ed..

[56] Alvarez S. (2015). Distortion Pathways of Transition Metal Coordination Polyhedra Induced by Chelating Topology. Chem. Rev..

[57] Gravogl L., Heinemann F.W., Munz D., Meyer K. (2020). An Iron Pincer Complex in Four Oxidation States. Inorg. Chem..

[58] Muthaiah S., Bhatia A., Kannan M., Muthaiah S., Bhatia A., Kannan M. (2020). Stability of Metal Complexes.

[59] Geng C., Li J., Weiske T., Schwarz H. (2018). Thermal O–H Bond Activation of Water As Mediated by Heteronuclear [Al2Mg2O5]•+: Evidence for Oxygen-Atom Scrambling. J. Am. Chem. Soc..

[60] Beck M.T. (1987). Critical Survey of Stability Constants of Cyano Complexes. Pure Appl. Chem..

[61] Dolomanov O.V., Bourhis L.J., Gildea R.J., Howard J.A.K., Puschmann H. (2009). OLEX2: A Complete Structure Solution, Refinement and Analysis Program. J. Appl. Cryst..

[62] Sheldrick G.M. (2015). SHELXT—Integrated Space-Group and Crystal-Structure Determination. Acta Cryst. A.

[63] Sheldrick G.M. (2015). Crystal Structure Refinement with SHELXL. Acta Cryst. C.

[64] Spek A.L. (2009). Structure Validation in Chemical Crystallography. Acta Cryst. D.

[65] Bell A., Aromí G., Teat S.J., Wernsdorfer W., Winpenny R.E.P. (2005). Synthesis and Characterisation of a {Ni8} Single Molecule Magnet and Another Octanuclear Nickel Cage. Chem. Commun..

[66] Aromí G., Bell A., Teat S.J., Winpenny R.E.P. (2005). Synthesis and Characterisation of a {Ni21Ag} Cage. Chem. Commun..

[67] Fitzpatrick A.J., Trzop E., Müller-Bunz H., Dîrtu M.M., Garcia Y., Collet E., Morgan G.G. (2015). Electronic vs. Structural Ordering in a Manganese (iii) Spin Crossover Complex. Chem. Commun..

[68] Boduszek B., Shine H.J. (1988). Preparation of Solid Thianthrene Cation Radical Tetrafluoroborate. J. Org. Chem..

[69] Chalkley M.J., Garrido-Barros P., Peters J.C. (2020). A Molecular Mediator for Reductive Concerted Proton-Electron Transfers via Electrocatalysis. Science.

