# Supplementary material for: Bis(2,6-pyrazolyl)pyridines as a New Scaffold for Coordination Polymers
Source: Molecules. 2023 May 23;28(11):4275. doi: 10.3390/molecules28114275 (PMC10254147; doi:10.3390/molecules28114275)
Supplement: Supplementary file 1 [file molecules-28-04275-s001.zip › molecules-2370378-supplementary.pdf]

Supplementary Materials

for

Bis(2,6-pyrazolyl)pyridines as a new scaffold for coordination polymers

Igor A. Nikovskiy[1,2], Pavel V. Dorovatovskii[3], Valentin V. Novikov[4*] and Yulia V. Nelyubina[1,2*]

Figure	Page number
**S1.** Plausible mechanism for the PCET-assisted formation of $[Fe(L^{OBF}{}_3)(CH_3COO)(CH_3CN)_2]_n{\bullet}nCH_3CN$.	3
**S2.** Reactant diffusion technique used for crystal growth.	3

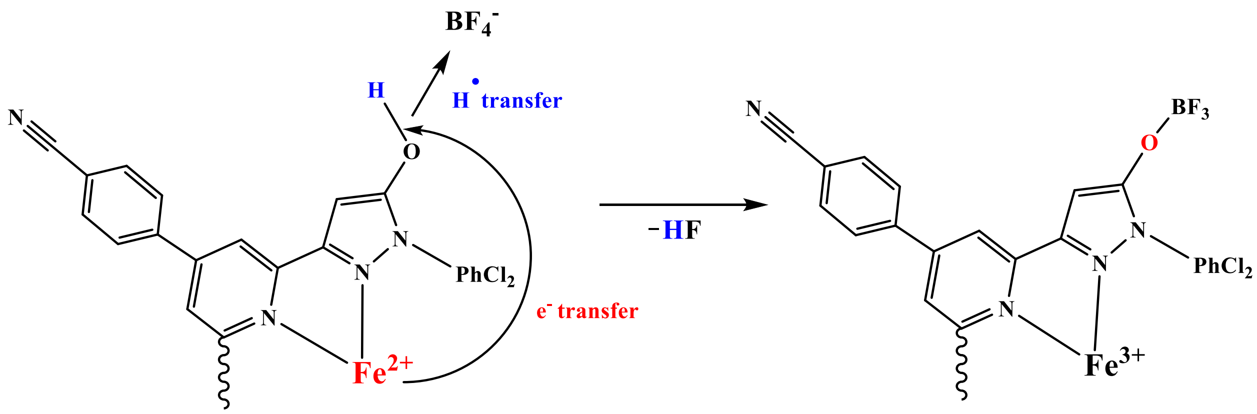

Figure S1. Plausible mechanism for the PCET-assisted formation of $[Fe(L^{OBF_3})(CH_3COO)(CH_3CN)_2]_n \cdot nCH_3CN$.

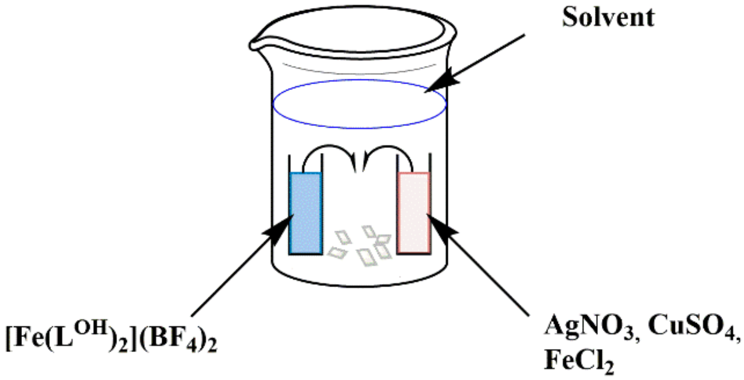

Figure S2. Reactant diffusion technique used for crystal growth.
